# An allosteric pathway explains beneficial fitness in yeast for long‐range mutations in an essential TIM barrel enzyme

**DOI:** 10.1002/pro.3911

**Published:** 2020-07-20

**Authors:** Yvonne H. Chan, Konstantin B. Zeldovich, Charles R. Matthews

**Affiliations:** ^1^ Department of Biochemistry and Molecular Pharmacology University of Massachusetts Medical School Worcester Massachusetts USA; ^2^ Program in Bioinformatics and Integrative Biology University of Massachusetts Medical School Worcester Massachusetts USA; ^3^ Sanofi Pasteur Cambridge Massachusetts USA

**Keywords:** allosteric pathway, biophysics, energy network, fitness, protein design, protein engineering, TIM barrel

## Abstract

Protein evolution proceeds by a complex response of organismal fitness to mutations that can simultaneously affect protein stability, structure, and enzymatic activity. To probe the relationship between genotype and phenotype, we chose a fundamental paradigm for protein evolution, folding, and design, the (βα)_8_ TIM barrel fold. Here, we demonstrate the role of long‐range allosteric interactions in the adaptation of an essential hyperthermophilic TIM barrel enzyme to mesophilic conditions in a yeast host. Beneficial fitness effects observed with single and double mutations of the canonical βα‐hairpin clamps and the α‐helical shell distal to the active site revealed an underlying energy network between opposite faces of the cylindrical β‐barrel. We experimentally determined the fitness of multiple mutants in the energetic phase plane, contrasting the energy barrier of the chemical reaction and the folding free energy of the protein. For the system studied, the reaction energy barrier was the primary determinant of organism fitness. Our observations of long‐range epistatic interactions uncovered an allosteric pathway in an ancient and ubiquitous enzyme that may provide a novel way of designing proteins with a desired activity and stability profile.

**Abbreviations**
CdRP: 1‐(o‐carboxyphenylamino)‐1‐deoxyribulose 5‐phosphate
I: intermediate state
IGP: indole‐3‐glycerol phosphate
IGPS: indole‐3‐glycerol phosphate synthase
*k*_cat_: rate constant
*K*_eff_: catalytic efficiency
*K*_m_: Michaelis–Menten constant
N: native state
*s*: selection coefficient
SCA: Statistical Coupling Analysis
ScIGPS: IGPS from *Saccharomyces cerevisiae*
SsIGPS: IGPS from *Sulfolobus solfataricus*
TCA: thermodynamic mutant cycle analysis
Trp: Tryptophan
U: unfolded state
*V*_max_: maximal rate of the reaction
*δ*: interaction energy
Δ*G*°: delta *G*, Gibbs free energy difference
ΔΔ*G*°: delta delta *G*, free energy difference between wildtype and mutant

## 1 INTRODUCTION

Proteins are molecular machines that catalyze chemical reactions required for metabolism, respiration, and many other processes essential for life. External stresses on a cell such as temperature, salt, or resource availability may require its constituent proteins to function at an altered rate or to develop divergent functions for survival.^1^ Neutral mutations from genetic drift could serendipitously result in a population of cells with enzymes capable of accommodating the selective pressure.^2^, ^3^, ^4^, ^5^, ^6^ Cells with beneficial mutations will, by definition, have greater reproductive success, that is, be more fit, under the external stress. Over time, the population of cells with enzymes capable of conferring enhanced fitness will increase under selective pressure and the allele become fixed.

**FIGURE 1:**
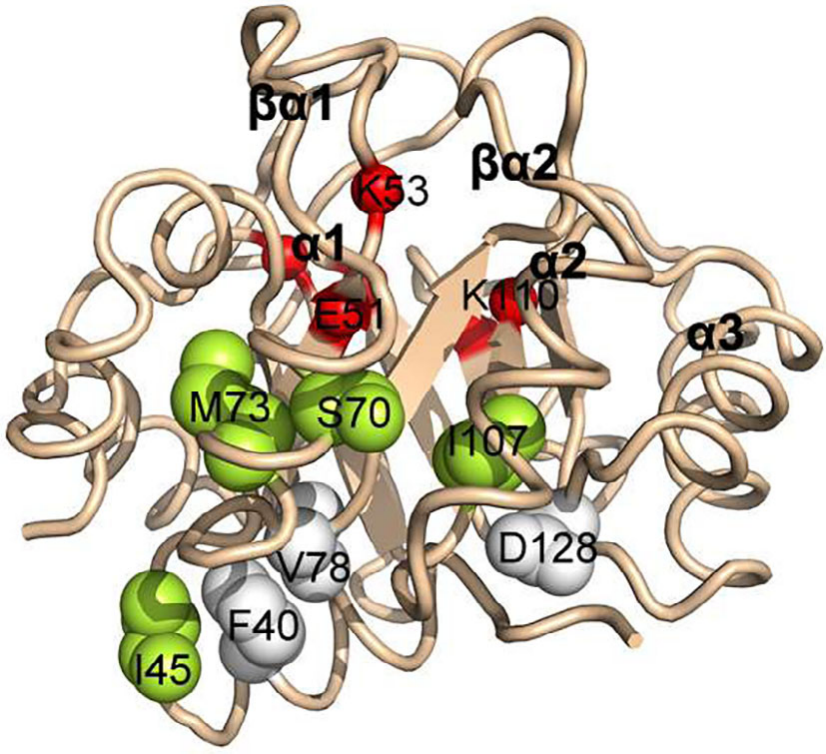
Wire diagram of SsIGPS, a canonical TIM barrel protein. Sites of mutations are highlighted in green with their wildtype side chains displayed as spheres. βα‐Hairpin clamp partners are highlighted in white with their wildtype side chains displayed as spheres. Active site residues are highlighted in red with the Cα displayed as spheres. (PDB: 2C3Z)

We have previously explored the relationship between genotype and phenotype with a yeast‐based competition fitness assay on an essential enzyme in the tryptophan (Trp) synthesis pathway, indole‐3‐glycerol phosphate synthase (IGPS).^7^ IGPS has a classic (βα)_8_ TIM barrel architecture,^8^, ^9^ and the phenotypic fitness response to saturating point mutations introduced into its stability elements, the αβ‐loops and β‐strands, was used to dissect the relationship with the genotypic expression of the sequence, that is, structure, stability, and function of the encoded protein. Although the majority of the mutations decreased fitness, we discovered that a set of mutations far from the active site resulted in a substantial increase in fitness^7^ (Figure 1). These increases were consistently observed across three thermophilic orthologs of IGPS, ensuring that the fitness effects were not specific to a particular IGPS sequence. We rationalized the relief of temperature stress on these thermophilic enzymes in a mesophilic host as a source of these beneficial mutations. However, we were quite surprised to observe allostery via this fitness assay. We and others^10^, ^11^, ^12^, ^13^ refer to the ensemble model^14^ of allostery, where a long‐range conformational change of thermodynamically coupled residues impact enzyme function caused by mutations. This effect is conceptually similar to the effect of ligand binding at a secondary effector site, the classic view of allostery described by Monod, Wyman, and Changeux.^15^, ^16^ IGPS catalyzes the conversion of the substrate CdRP to the product IGP, independent of other enzymes in the Trp synthetic pathway, obviating a role for allostery in its function.

Examination of the competition fitness data revealed that the greatest fitness was associated with mutations at the N‐terminal end of the β‐barrel, opposite the active site cradled in the βα‐loops at the C‐terminal end (Figure 1).^7^ Another set of beneficial mutations was found for residues in the β‐strands whose side chains extend out from the barrel and provide docking sites for the α‐helical shell. By contrast, replacements of side chains in β‐strands that protrude into the interior of the β‐barrel were almost uniformly deleterious. Taken together, we hypothesized that the allosteric coupling between the N‐terminus of the β‐barrel and the active site is mediated by the α‐helical shell.

To test this hypothesis, we constructed a set of single and double mutations between a highly beneficial site at the N‐terminus of the β‐barrel and a pair of sites in helix α1 that transverses the β‐barrel. Quantitative assessments of the perturbations in the stability and catalytic properties were the input for the thermodynamic mutant cycle analysis (TCA)^17^ to test for interactions between the sites. Additive perturbations when comparing the combined effects of the single mutants and those of the double mutant are indicative of independence, while nonadditivity is indicative of interactions between the pair of amino acids. We found evidence for nonadditivity between the N‐terminus of the β‐barrel and the α‐helical shell of the IGPS from *Sulfolobus solfataricus* (SsIGPS), supporting the hypothesis that the shell is the conduit for allostery in this TIM barrel enzyme. This ancient and ubiquitous fold serves as a robust platform for understanding protein evolution, function, folding, and design.

## 2 RESULTS

### 2.1 *General considerations*

Mutations can have large effects on protein structure, stability, and function. Thus, we considered five parameters in our genotype to phenotype analysis of our point mutants in SsIGPS: organismal fitness, intracellular protein abundance, protein stability, structure, and catalytic efficiency. At the macroscopic phenotypic level, organism fitness is expected to reflect the integration of the four microscopic biophysical and biochemical properties. The choice of positions to test for allostery was guided by the results of the previous saturation mutagenesis experiments and the functional sector identified by the Statistical Coupling Analysis (SCA) of 537 IGPS sequences.^7^, ^18^ We chose to focus on residue I45 that forms part of a noncanonical βα‐hairpin clamp with F40 and V78 (Figure 1). Mutation of I45 to all other amino acid resulted in beneficial fitness effects,^7^ presumably reflecting disruption of the clamp. The choice of I45 as a target of our study was also supported by our SCA, which identified two significant sectors—a “stability” Sector 1 and a “functional” Sector 2 (see Reference 7). As previously shown, Sector 1 includes the hydrophobic side chains between the β‐barrel and the α‐helical shell, essentially defining the TIM barrel architecture. Sector 2, in turn, identified all of the essential residues in the active site, and, remarkably, a string of residues in the α‐helical shell that lead to I45.

Alanine was chosen as the standard for mutations because its C_α_ atom enforces the main chain energy surface, that is, the Ramachandran map, and its small size avoids steric clashes. Mutations to lysine were also examined because these cationic side chains had a striking beneficial fitness in the mutagenesis assay. To probe the putative pathway for allostery, we examined the coupling between beneficial I45 (functional Sector 2) mutations and two positions in helix α1, S70 (stability Sector 1) and M73 (functional Sector 2), bridging the noncanonical βα‐hairpin clamp and the active site. The distances between the Cα carbons are 17 and 24 Å for I45 to the closest catalytic side chains, E51 and K53, respectively. Residues S70 and M73 are 18 and 14 Å away from I45, and 9–13 Å from the E51 and K53 side chains. We also examined residue I107 that participates in a canonical βα‐hairpin clamp with D128, functionally analogous to the hydrophobic clamp that I45 participates in. While D128 was found to be in Sector 2, I107 was not, highlighting the unique properties of SsIGPS structure as compared to the global couplings in the TIM barrel sequence alignment.

### 2.2 *Thermodynamic mutant cycle analysis*

The interaction or deviation from additivity between any given two sites, *δ*, for a physical property (*P*) is calculated by the following equation^17^:

$$\delta =\Delta P_{\mathrm{DM}}–\left(\Delta P_{\text{M1}}+\Delta P_{\text{M2}}\right)$$

where Δ*P*_M1,2_ = *P*_M1,2_ – *P*_WT_, and *P*_WT_, *P*_DM_, *P*_M1_, and *P*_M2_ are quantitative measures of the property of the wildtype protein, the double mutant, *P*_DM_, and the pair of single mutants, *P*_M1_ and *P*_M2_. Complete additivity would result in *δ* = 0, implying no interaction. A nonzero *δ* indicates epistasis.

To apply the TCA, we created a set of double mutants to test the additivity of the interactions between two sites. The double mutants, I45A/S70A, I45K/S70A, I45A/M73A, are found within the β1α1β2α2 module^19^ of the TIM barrel and enabled a test of the interactions between the noncanonical βα‐hairpin clamp^20^ and the intervening α1 helix. To test if βα‐hairpin clamps between modules interact, we studied a pair of double mutants, I45K/I107A and I45K/I107K. I107 donates its main chain NH to a classical βα‐clamp with the aspartic acid side chain, D128. Lastly, we tested the long‐range interaction between residues in the α‐helix of the module β1α1β2α2, to the βα‐hairpin clamp of the adjacent β3α3β4α4 module with the double mutants, S70A/I107K and M73A/I107K. Unfortunately, attempts to purify M73A/I107K for biophysical and biochemical characterization resulted in a low yield, precluding a study of this mutant. A table of the 12 mutations, including a rationale for their role in testing the TCA is provided (Table S1).

### 2.3 *Fitness*

Fitness refers to the growth rate, or the inverse of the doubling time for tryptophan auxotrophic IGPS knockout yeast, *Saccharomyces cerevisiae*, transformed with the mutant SsIGPS construct. The relative fitness was quantified with the selection coefficient, *s*, which compares the growth rate constant, *k*, of the mutant to our SsIGPS wildtype (SsWT) on a log_2_ scale, *s* = log_2_(*k*_MT_/*k*_WT_). A mutant with a selection coefficient of 0 has a comparable growth to SsWT, whereas a selection coefficient of −1 has a comparable lethal phenotype to null or uncomplemented yeast. Yeast with growth rate constants exceeding SsWT have selection coefficients greater than 0 and are considered to harbor beneficial mutations.

**FIGURE 2:**
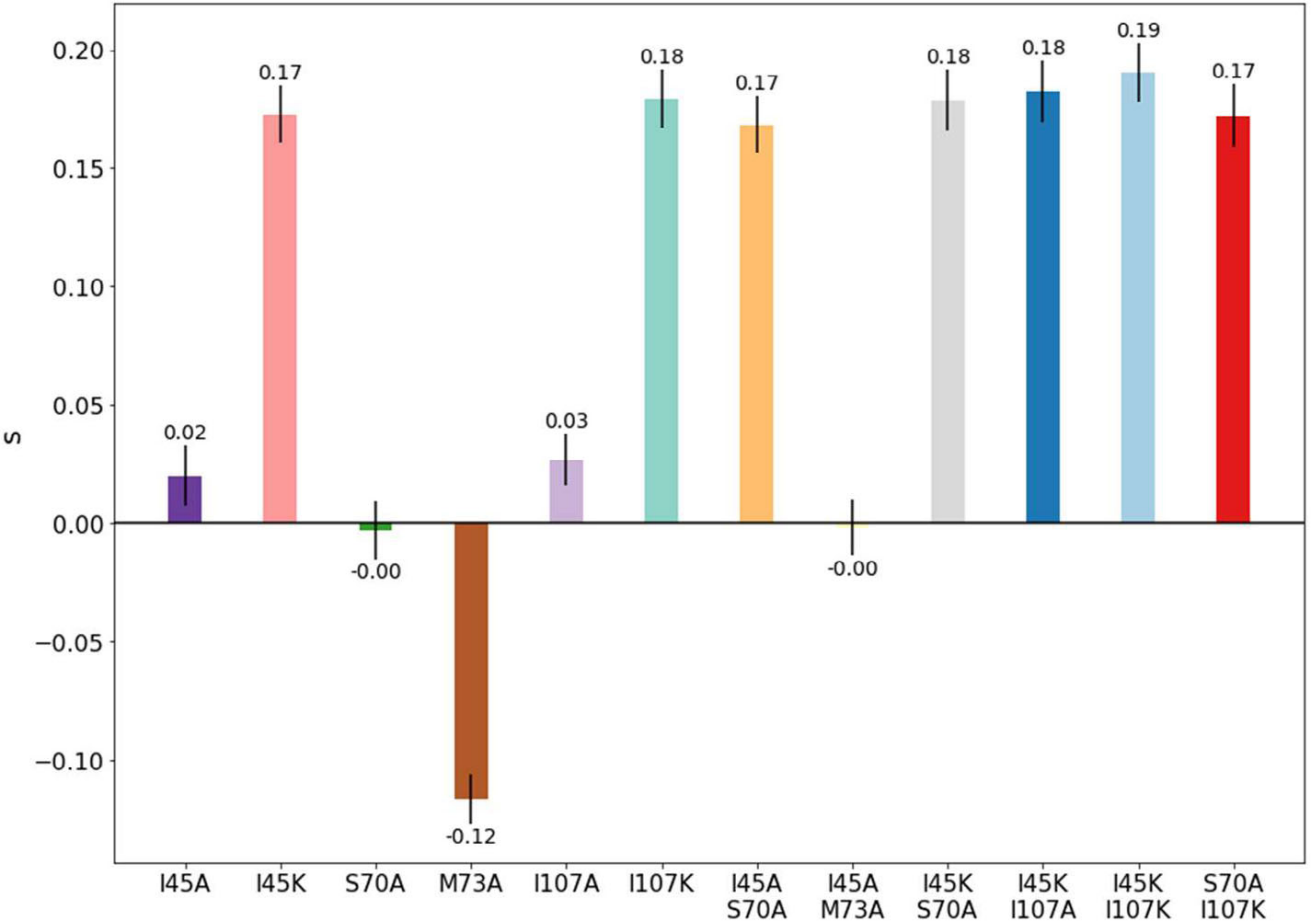
Selection coefficients of single and double mutants in SsIGPS. Selection coefficients are plotted on a bar graph to show the relative fitness of the mutants to wildtype. Bars are colored qualitatively to distinguish mutants. Most mutations are neutral (*s* ~ 0) or beneficial (*s* > 0). Selection coefficients for beneficial mutations plateaus around *s* ~ 0.18. Vector‐only controls (not shown) result in lethality (*s* ~ −1). Errors are propagated from the standard deviation of measurements from three biological samples

All mutations in this study conferred wildtype‐like (*s* ~ ±0.03) or beneficial fitness, except M73A which is slightly deleterious. The selection coefficients range from −0.12 for M73A to 0.19 for I45K/I107K, with the beneficial I45K, I107K, I45A/S70A, I45K/S70A, I45K/I107A, I45K/I107K, and S70A/I107K mutants all having *s* values of 0.18 ± 0.01 (Figure 2, Table S1). The significant differences in the *s* values for I45A and I45K, 0.02 and 0.17, demonstrate the sensitivity of the growth rate to the chemical properties of the side chain. Positional sensitivity is also evident. The S70A mutation resulted in neutral fitness (*s*_S70A_ = 0.00), while one turn downstream, the M73A mutation resulted in poorer fitness (*s*_M73A_ = −0.12). We also compared the effects of mutating the noncanonical βα‐hairpin clamp involving I45 to that of a canonical clamp involving I107. Analogous to I45, I107K produced a notable fitness enhancement, while the I107A mutation produced a more modest effect (*s*_I107K_ = 0.18, *s*_I107A_ = 0.03). The very similar selection coefficients for I45K, I107K, and, with the exception of I45A/M73A, all 5 remaining double mutants, *s* = 0.18 ± 0.01, demonstrate the potential for a plateau in beneficial effects for mutations that disrupt both the β1α1β2α2 and the β3α3β4α4 clamps (Figure 2). The unanticipated saturating effect of the I45K and I107K mutations and double mutations involving them precludes the application of the TCA to fitness.

### 2.4 *Intracellular protein abundance*

For the growth assay and protein quantification, three biological replicates for each IGPS variant were measured. We found that mutant protein abundances were 1 to 2.5‐fold to that of SsWT by Western blot analysis (Table S1). The observation of mutant proteins with higher concentrations than WT protein is most likely explained by the limitations in the sensitivity of our detection assay. Since protein expression is induced constitutively in our system and there is no feedback or derepression mechanism by the host on IGPS production (see Methods), we would expect the concentrations of the mutant proteins to be comparable to or less than WT. The apparent ~2‐fold lower concentration of WT SsIGPS would only serve as a consistent offset in the selection coefficients because they are normalized against the growth rate constant for WT SsIGPS.

### 2.5 *Global secondary structure*

**FIGURE 3:**
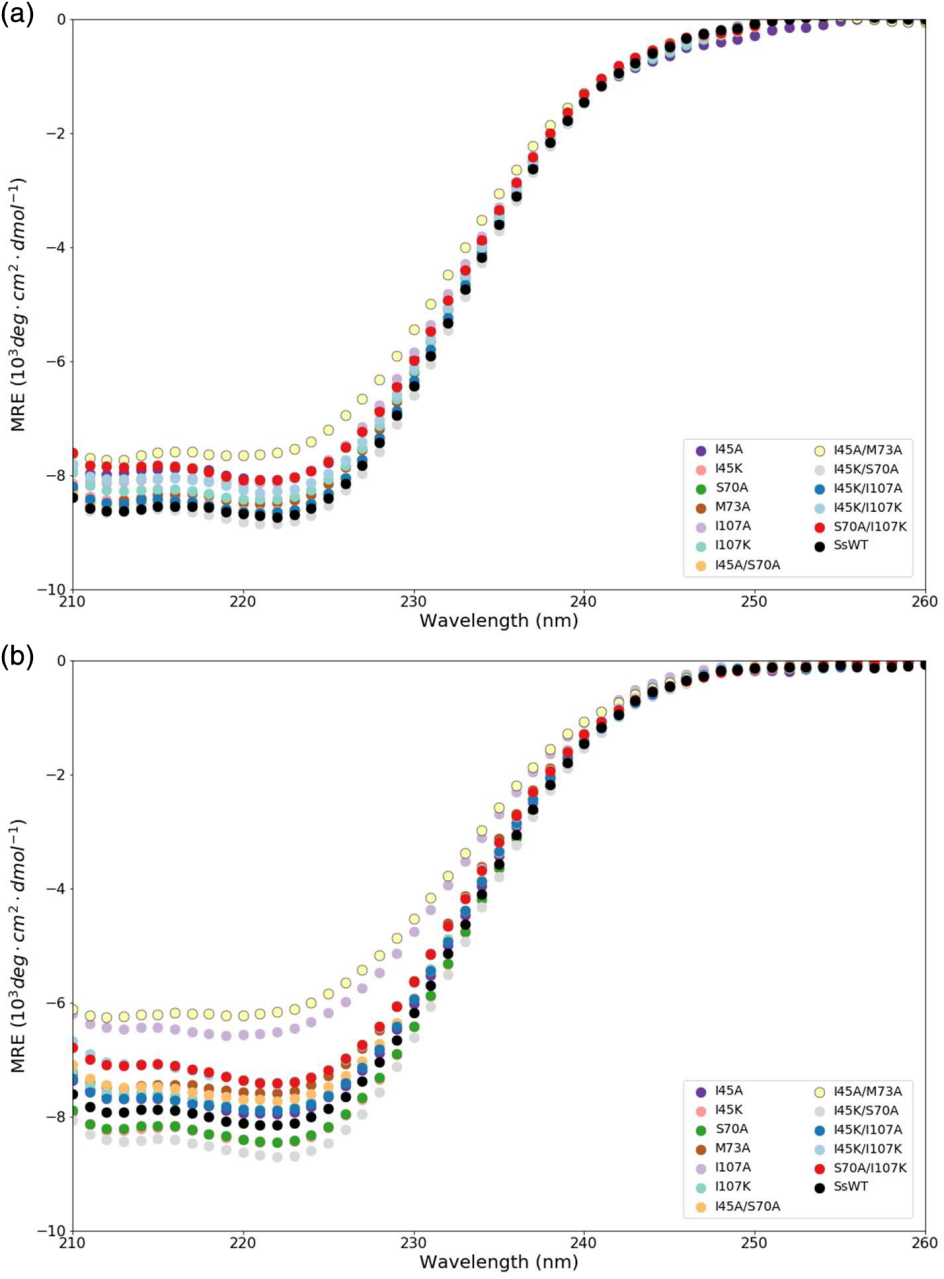
Far‐UV mean residue ellipticities of WT and mutant SsIGPS. Far‐UV CD spectra of SsWT and mutants collected at (a) 25°C (b) 30°C in 10 mM KPi, pH 7.2. For reference, the SsWT spectrum is shown in black. Mutant spectra are shown using a qualitative color palette. At both temperatures, the prominent negative band at 222 nm is indicative of the presence of α‐helix secondary structure. At 30°C, reduced CD signals for I107A (light purple) and I45A/M73A (yellow) suggest the proteins are partially unfolded

The secondary structure of the SsIGPS mutants was monitored by far‐UV CD spectroscopy (Figure 3a). The ellipticities of the mutant proteins are comparable to the WT protein and have the characteristic minimum at ~222 nm for βα‐repeat proteins, ensuring that all achieve the native TIM barrel fold at 25°C. The reduced ellipticities for the I107A and I45A/M73A at 30°C (Figure 3b) reflect their significantly reduced stabilities to urea denaturation (see below) and partial unfolding at 30°C.

### 2.6 *Protein stability*

**FIGURE 4:**
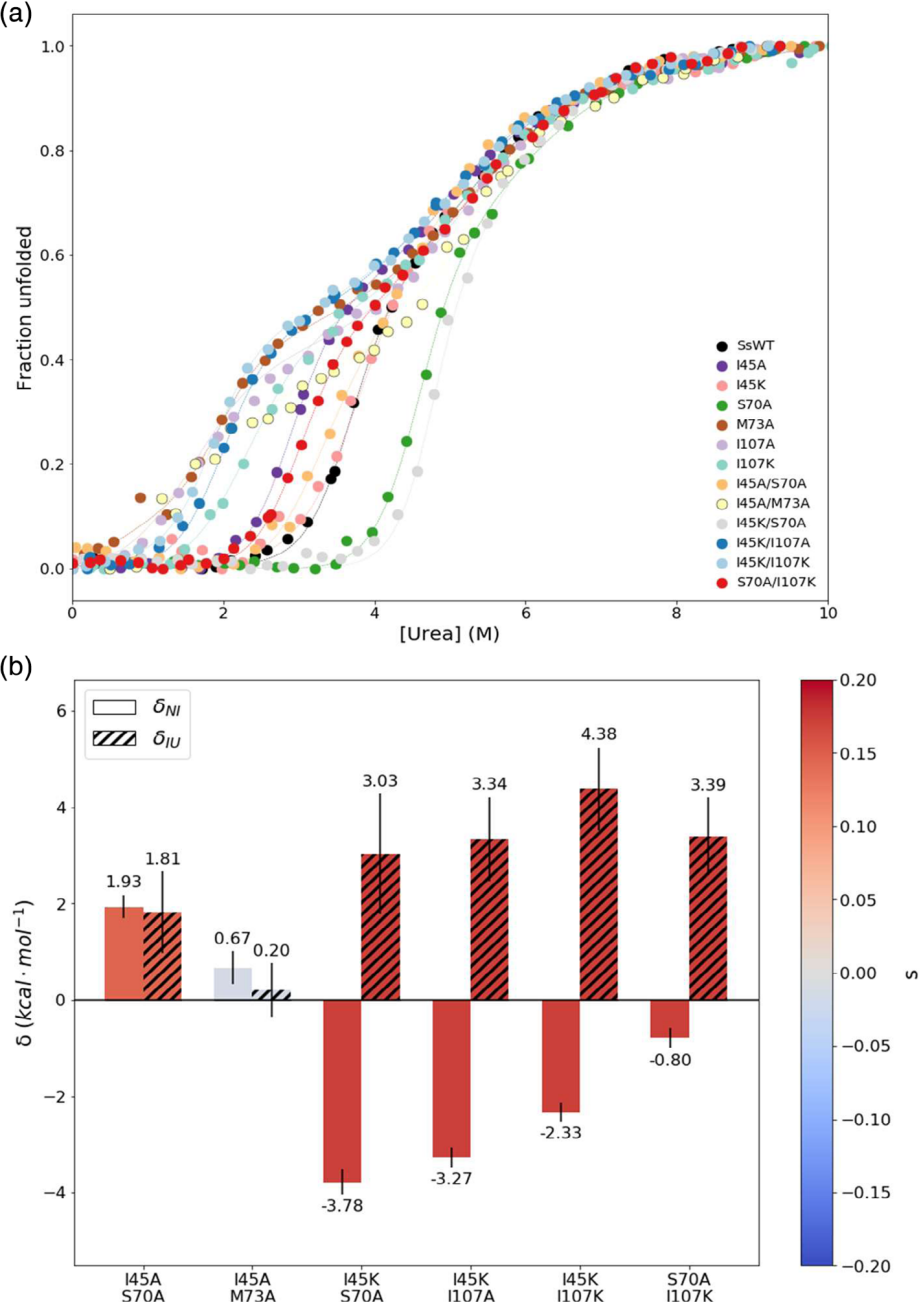
Protein stabilities and interaction energies of WT and mutant SsIGPS. The entire CD spectra as a function of urea for each variant were globally fit to a three‐state model and the results plotted as fraction unfolded. (a) Collected sample reads are indicated by the filled circles. Fits to the data are indicated by the dash lines. Urea melts observed by CD at 222 nm show stabilities of mutants differed from SsWT for both the native and intermediate states. Mutant titrations are shown using a qualitative color palette. (b) Interaction energies, *δ*_NI_ and *δ*_IU_, were calculated using the ΔΔ*G* values for both the NI and IU transitions. All double mutants show nonzero interaction energies. Bars are colored by selection coefficients indicated by the color scale. Errors were propagated from the fit of the model

Equilibrium urea denaturation curves displayed a three‐state unfolding process for all variants examined: Native (N) ⇌ Intermediate (I) ⇌ Unfolded (U) (Figure 4a). All of the single and double mutations significantly perturb the free energy difference between the N and I states (Figure S1, Table S2). The S70A and I45K/S70A mutations increased the free energy difference, while all others decreased the free energy difference between N and I states. Effects on the I/U transition vary; five mutants showed a noticeable increase in Δ*G*_IU_: I45K, S70A, M73A, I45A/M73A, and I45K/S70A, while the remainder had comparable stabilities.

Application of the TCA to the urea denaturation data revealed that *δ*_NI_ and *δ*_IU_ for the I45A/S70A, I45K/S70A, I45K/I107A, and I45K/I107K double mutants were nonadditive; the small magnitudes of the *δ* values for I45A/M73A in both transitions and S70A/I107K for the N/I transition preclude definitive conclusions about additivity. (Figure 4b, Table S3). These results demonstrate that I45 of the noncanonical hairpin clamp between β1 and β2 interacts with S70A and, possibly, M73 in α1, as well as the canonical hairpin clamp (I107) between β3 and β4.

### 2.7 *Enzymatic activity*

**FIGURE 5:**
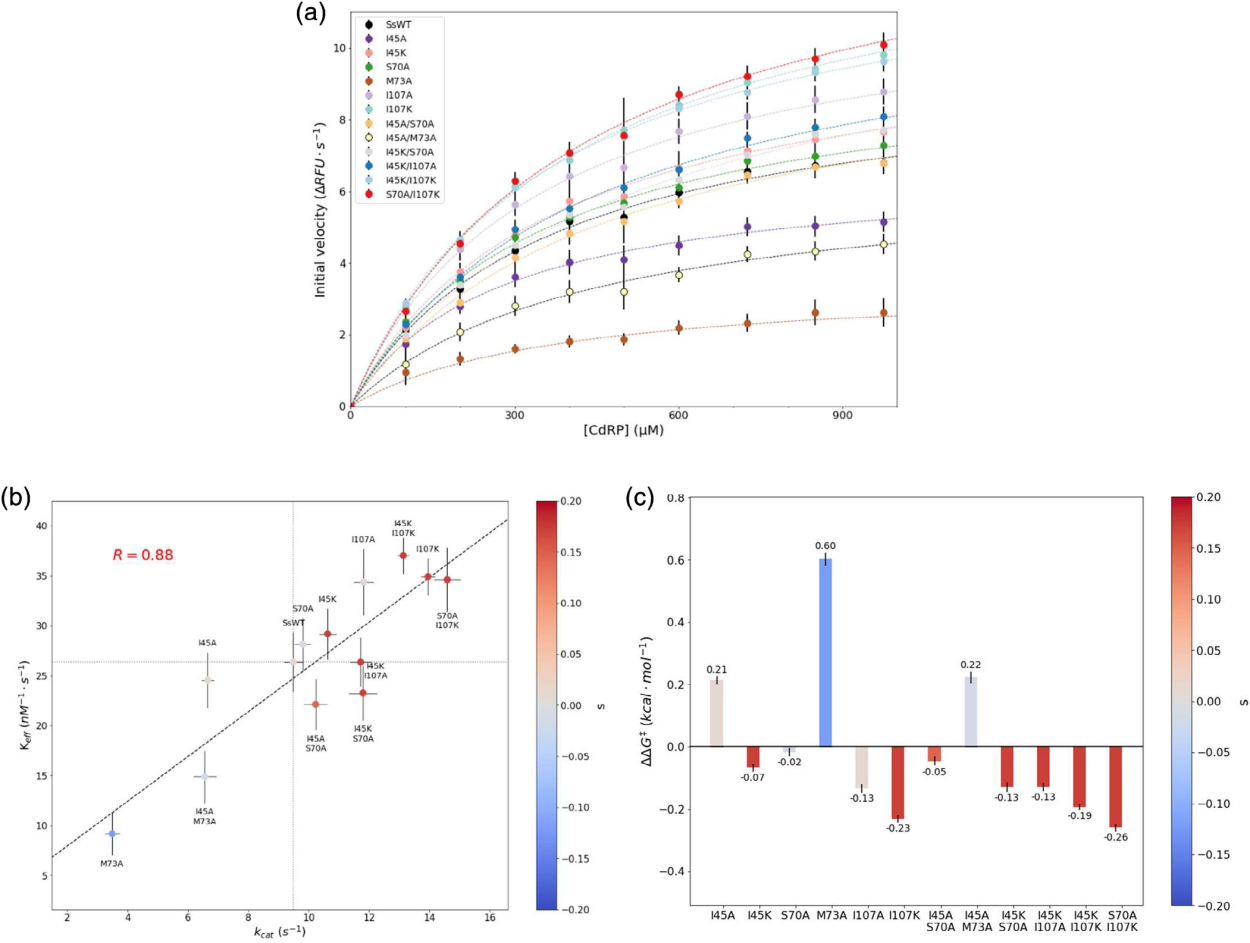
Catalytic properties of WT and mutant SsIGPS enzymes. (a) Initial velocity measurements were collected to determine the catalytic efficiency of each SsIGPS variants at 30°C in 10 mM KPi, pH 7.2. Fluorescence readings from the product formation (circle marker) were fit to the Michaelis–Menten equation (dashed line) to determine the kinetic parameters, *k*_cat_, *K*_m_, and *k*_eff_. Markers are colored qualitatively to distinguish mutants. (b) The *K*_eff_ was plotted as a function of *k*_cat_ for each of the SsIGPS variants. A positive linear relationship was observed between the turnover number and the catalytic efficiency. Markers are colored by selection coefficient. Mutants are individually labeled near the marker. The fit is denoted by the black dashed line. The gray dotted lines are visual guides for the values associated with SsWT. (c) Interaction energies, *δ*_kcat_, were calculated using the activation energy barrier energy, ΔΔ*G* ^‡^ values for the *k*_cat_ transitions. All double mutants show nonzero interaction energies. Bars are colored by selection coefficients indicated by the color scale. Errors are propagated from the standard deviation of measurements collected from three biological samples

An important metric for assessing the molecular basis for the phenotypic effects of mutations on fitness is the effect on catalytic properties. Kinetic parameters were determined for the conversion of CdRP to IGP by IGPS. Initial velocities of each of the SsIGPS variants were measured at multiple concentrations of substrate to derive *V*_max_, *k*_cat_, *K*_m_, and the catalytic efficiency *K*_eff_ = *k*_cat_/*K*_m_, using the Michaelis–Menten formalism (Figure 5a). The *k*_cat_ values varied from 3.5 s^−1^ for M73A to 14.6 s^−1^ for S70A/I107K, slower and faster than the WT *k*_cat_ of 9.5 s^−1^ (Figure S2, Table S2). By contrast, the *K*_m_ values varied over a narrower range, 271–507 μM, bracketing the WT *K*_m_ of 359 μM. The *K*_eff_ values fell into three classes: those with WT‐like *K*_eff_, I45A, I45K, S70A, I45A/S70A, and I45K/I107A, those with greater *K*_eff_ I107A, I107K, I45K/107 K, and S70A/I107K and those with smaller *K*_eff_, M73A and I45A/M73A. The linear correlation between *K*_eff_ and *k*_cat_ (Figure 5b), *R* = 0.88, demonstrates that the major effects of the mutations are on *k*_cat_.

The catalytic rate constants for SsWT and the mutant proteins, *k*_cat_, can be used to calculate the perturbation in the activation energy barrier, ΔΔ*G* ^‡^, via *k*_cat_ = *k*_0_e^−Δ*G*‡RT^ (Figure 5c, Table S2).^21^ A negative value in ΔΔ*G* ^‡^ indicates a lower energy barrier associated with the mutation. The nonzero interaction energy, *δ*, for the I45A/S70A and I45A/M73A double mutants revealed allostery between the noncanonical clamp at the N‐terminus of the β‐barrel and the two positions on the hydrophobic face of α1. Individual mutations at both sites affect *k*_cat_, but their mutual interaction causes a nonadditive effect on *k*_cat_ for the double mutant. The small magnitude for the *δ* values for the I45K/S70A, I45K/I107A, I45K/I107K, and S70A/I107K demonstrate sequence specificity (I45K/S70A), but do not provide definitive evidence for interactions between the two N‐terminal clamps that perturb *k*_cat_.

## 3 DISCUSSION

Organism fitness is subject to the local environment. A beneficial phenotype in one setting may be an impediment in another, varying the effect of fitness and the strength of selection.^22^ While SsIGPS can complement the knockout condition and prevent lethality in synthetic *‐trp* drop‐out media, the fitness is lower than when the endogenous *S. cerevisiae* IGPS gene (ScIGPS) is reintroduced using the same complement system. The doubling time of yeast with ScIGPS is ~3 hr versus ~4–5 hr for WT SsIGPS. Despite sharing a common structure and function, the increased doubling time shows that the hyperthermophilic SsIGPS enzyme is not optimized for the mesophilic temperature of the yeast, creating an opportunity for mutation to improve fitness. The appearance of beneficial mutations far from the active site in SsIGPS provides prima facie evidence for the allosteric interactions explored in the present study.

### 3.1 *Molecular basis for beneficial mutations*

The molecular basis for the enhanced fitness observed in beneficial mutations can be understood in terms of the catalytic mechanism for IGPS. The chemical process is the same for the yeast and prokaryotic orthologs; however, the rate limiting step in catalysis differs.^23^ For SsIGPS, the rate determining step for catalysis at thermophilic temperatures is temperature‐dependent.^23^ Long loops at the C‐terminus of the β‐barrel protect the active site from the solvent environment, but also play a role in catalysis.^24^ The interaction between β1α1 and β2α2 loops has been shown to have functional consequences including substrate binding, chemistry, and product release.^24^ At mesophilic temperatures, these two long loops are less flexible and strong electrostatic interactions between K53 and the C1 and C2′ of CdRP result in an unproductive extended substrate conformation.^25^, ^26^ At higher temperatures, an enhanced population of K53 conformers is found to have a greater distance between K53 and the substrate, resulting in a productive orientation of CdRP.^25^, ^26^ The modulation of this structurally mediated rate limiting step at mesophilic temperatures is driving the beneficial fitness response in this thermophilic enzyme.

**FIGURE 6:**
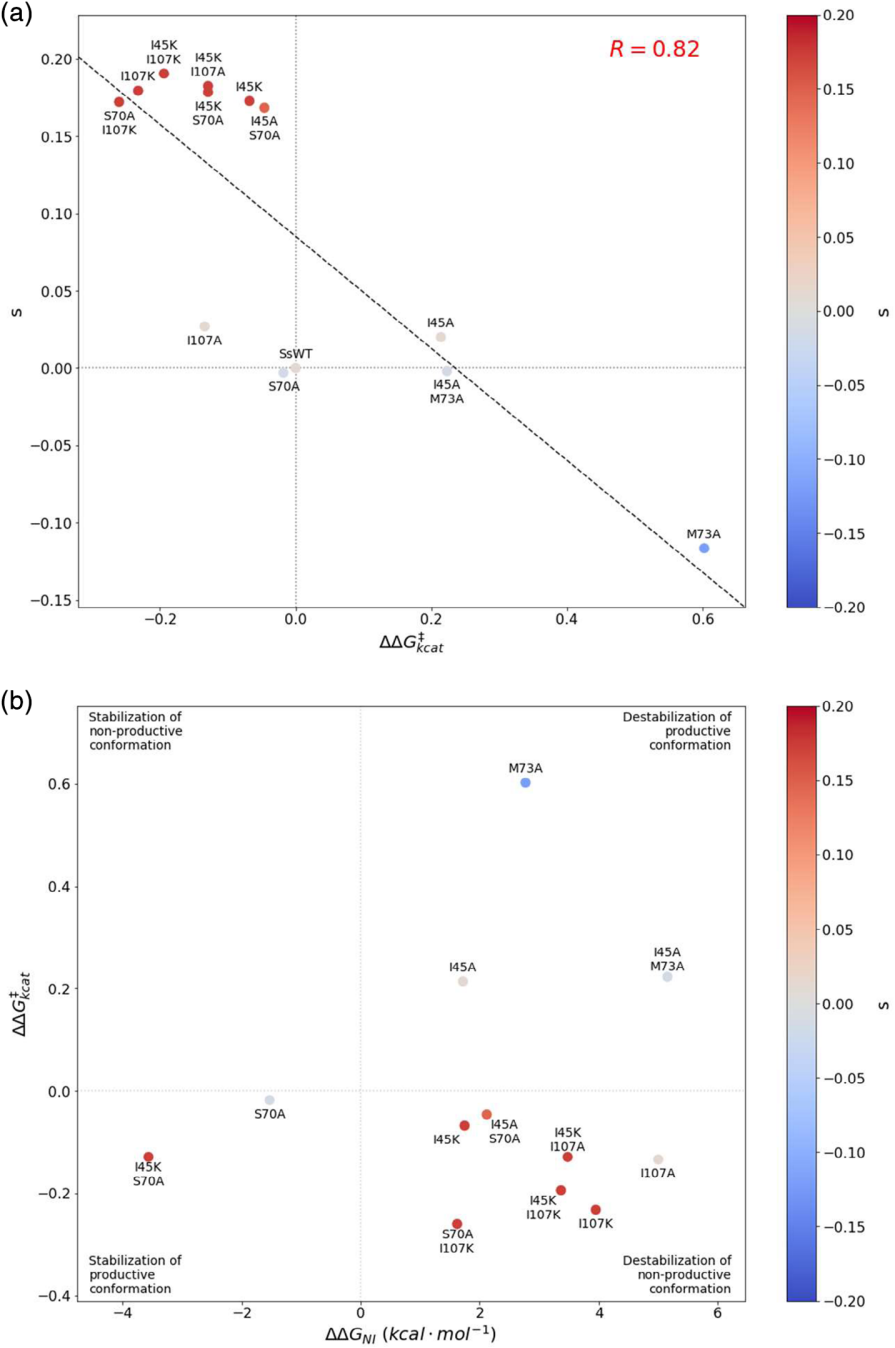
Correlation of selection coefficients and activity and stability‐activity plot for WT and mutant SsIGPS. (a) Selection coefficient is negatively correlated with transition state activation energy of catalysis. Values of *s* were plotted as a function of ΔΔ*G* ^‡^_kcat_ for each of the SsIGPS variants and showed a negative linear relationship. The fit is denoted by the black dashed line. Markers are colored by selection coefficient. The gray dotted lines are visual guides for the values associated with SsWT. (b) Fitness landscape of mutants on function‐stability energy plane. Markers are colored by selection coefficient. Within the native well of the folding free energy surface, a complex relationship is found between activity and stability. No mutants are found on the upper left quadrant of the energy plane in our experiments

It is interesting to observe a maximum plateau in the fitness of single and double mutant proteins at *s* = 0.18 (Table S1, Figure 2). This response may stem from either (a) epistatic sites optimizing the same mechanism and/or (b) the minimum threshold of tryptophan required for optimal growth has been met and no additional fitness advantage is gained by additional tryptophan synthesis. Hartl et al. have noted that for intermediate metabolic enzymes, there is a saturation point of “diminishing returns,” where increased throughput yields minimal increases in fitness.^27^ While both substrate turnover and binding affinity can affect catalytic efficiency, we observed a strong positive correlation between selection coefficient and ΔΔ*G* ^‡^_Keff_ (*r* = 0.65), but an even stronger one between selection coefficient and ΔΔ*G* ^‡^_*k*cat_ (*r* = 0.82) (Figure 6a). These findings suggest that substrate turnover is driving the beneficial fitness effects.

Allosteric interactions in the TIM barrel structure resulted in a complex fitness landscape in the stability‐activity plane, parametrized by ΔΔ*G*°_NI_ and ΔΔ*G* ^‡^_*k*cat_. High fitness mutations are characterized by the lower activation energy and increased catalytic activity (negative ΔΔ*G* ^‡^_*k*cat_ values) (Figure 6b). Most of the high fitness mutations also destabilized the protein (positive ΔΔ*G*°_NI_ values), consistent with adaptation of a hyperthermophilic enzyme to mesophilic conditions (Figure 6b, lower right quadrant). The largely positive ΔΔ*G*°_NI_ (destabilized native state) and lower activation energy ΔΔ*G* ^‡^_*k*cat_ associated with higher fitness suggest beneficial mutations may be destabilizing the nonproductive conformation (lower right quadrant), whereas neutral or deleterious mutations may be destabilizing the productive conformation (upper right quadrant). We also observed a case, I45K/S70A, where the mutations may be stabilizing the productive conformation (lower left quadrant). In our limited sampling, no examples were found for a mutant that is more stable and had a greater activation energy (upper left quadrant).

Flux dynamics theory is used to describe fitness as a function of the effective functional capacity, which takes into account both functional protein abundance and catalytic efficiency.^28^, ^29^, ^30^ In Figure S3, we tried to fit our data to the flux balance model. Consistent with previous models,^29^ we observed a positive trend between fitness, abundance of functional protein, and catalytic activity. However, this observation is limited by uncertainty in the measurement of intracellular protein concentration.

### 3.2 *Allostery in TIM barrel proteins*

The input data for the TCA analysis of intramolecular interactions, Δ*G*°_NI_ and *k*_cat_, both support the transmission of structural and energetic information from the N‐terminus of the β‐barrel to the distal active site via the α‐helical shell. This allosteric pathway is reminiscent of the one reported for the analogous imidazole glycerol phosphate synthase (ImGPS) that catalyzes the fifth step in the histidine pathway.^31^, ^32^ The heterodimeric ImGPS protein, composed of HisF, a TIM barrel, and HisH, is regulated by a V‐type (*k*_cat_, not *K*_m_, driven) mechanism where the glutamine amidotransferase activity of HisH is activated only upon the binding to the effector region on HisF.^32^, ^33^ Upon substrate binding, structural rearrangement of HisF starts from the catalytic βα‐loop1 and propagates down the β‐barrel through residues along β2, α2, and α3, to the distal end, altering the HisF/HisH interface and activating HisH.^32^, ^33^ To our knowledge, ImGPS is the only TIM barrel with a naturally occurring allosteric regulatory mechanism.

The HisF/HisH example clearly demonstrates the potential for allosteric signal propagation through energetically linked residues along the β‐barrel/α‐helical interface, as observed with our beneficial mutations and TCA analysis of IGPS. Interestingly, temperature has a pronounced effect on the allosteric mechanism of thermophilic *Thermotoga maritima* ImGPS, where allosteric activation of HisH by HisF is significantly increased at 30°C compared to 70°C. The authors attributed this activation to alternative communities of residues that are formed in a temperature dependent manner.^34^ In our study, mutations might induce corresponding changes that manifest themselves in the observation of allostery. Detailed simulations of the SsIGPS mutant proteins from our study, focusing on the coupling of the structure and dynamics of the active site with the global dynamics of the TIM barrel, would provide further mechanistic insights into its allosteric pathways.

### 3.3 *Implications for the de novo design of TIM barrel enzymes*

TIM barrel enzymes are involved in numerous essential metabolic pathways and are capable of catalyzing six of seven fundamental chemical reactions.^35^ The potential of this platform to transform unnatural substrates to valuable products has motivated the de novo design of a novel TIM barrel enzyme.^36^ Supposing that stabilizing mutations outside the active site promote evolution,^37^, ^38^, ^39^ the canonical βα‐hairpin clamps in TIM barrel enzymes may have provided the necessary stability to adapt biochemical activity to the environment.^40^, ^41^ Our results suggest there is a fine balance between stability and activity, where stabilization by β1α1‐hairpin clamp can negatively influence enzyme function under cold temperature stress due to long‐range interaction between the hairpin clamp and the active site. Great strides have already been taken with the first successful fourfold, fully symmetric TIM barrel based on carefully ascribed rules for geometric constraints.^36^ However, incorporating specific biochemical function, even using naturally occurring scaffolds, remains a challenge with the requirement to include dynamics and multiple conformations in the design, for example, the unexpected conformational rearrangements of the active site loops.^42^

## 4 CONCLUSIONS

Rather than selecting for novel function, we challenged three thermophilic or hyperthermophilic TIM barrel enzymes to perform in mesophilic temperatures.^7^ While functional, fitness was reduced by an overly stable nonproductive conformation at the nonpermissive temperature.^43^, ^44^ By sampling and modulating the energy landscape between stability and activity in SsIGPS, we identified mutations that rescued the yeast host from the cold stress. In the process, we uncovered a latent allosteric pathway in a widely used and well‐studied system. The ability to selectively stabilize the productive loop conformation through knowledge of the underlying “wiring”^45^ of the IGPS energetic network offers the potential to control the enzyme activity. Through this work, we identified an allosteric pathway from the canonical βα‐hairpin clamp region to the active site via the α‐helical shell that increased the catalytic efficiency of a cold‐stressed TIM barrel enzyme and improved the fitness of the auxotrophic host. A mechanistic understanding of how residues within a TIM barrel are energetically interconnected opens the possibility of engineering conformational control for specific functions onto a de novo TIM barrel via first principles. Introducing allostery in the design algorithm would be a major step toward designing “fit” and functional proteins.

## 5 MATERIALS AND METHODS

### 5.1 *Yeast strain and culture conditions*

*S. cerevisiae* strain BY4742 Δ*IGPS::KanMX* was produced using the same PCR‐generated deletion strategy described by the Saccharomyces Genome Deletion Project.^46^ The last 810 bp of the TRP3 gene encoding IGPS were replaced with the KanMX gene. Deletion of IGPS with the KanMX gene was confirmed by Sanger sequencing.

The same expression vector used in the previously described EMPIRIC fitness assay was used for the individual growth assays. The pRS416 vector carrying the auxotrophic URA3 marker was a gift from Professor Daniel Bolon's laboratory at the University of Massachusetts Medical School. A lower expressing constitutive Tma19 promoter, also provided by the Bolon laboratory, was used to increase the sensitivity of the fitness assay. Three silent mutations were introduced into the plasmid at the URA3 marker, the ampicillin resistance marker, and the Tma19 promoter to disrupt BSAI recognition sites. The BSAI enzyme was used to create the saturating mutagenesis libraries.

The wildtype SsIGPS gene was purchased from Genscript. An N‐terminal 6× His tag, and Tev protease recognition site were added for protein purification and protein abundance measurements. The noncanonical N‐terminal α00 (residues 1–26) of each gene was deleted to reduce aggregation during refolding of purified proteins. To prevent nonspecific cleavage when using Tev protease, position 18 was mutated from arginine to serine. Point mutations were introduced into the wildtype background through site‐directed mutagenesis using the recommended PCR protocol for Phusion® High Fidelity DNA polymerase.

Yeast transformation was performed using the LiAc/SS carrier DNA/PEG method as described by Gietz and Schiestl.^47^ Yeast cells were grown in rich media with G418 to select for IGPS knockout yeast. Transformed cells were selected on synthetic minimal media lacking uracil. Selection for IGPS activity was achieved through growth of transformed yeast (one plasmid library per culture) in synthetic drop‐out medium lacking tryptophan. All growth experiments were performed at 30°C. Liquid cultures were maintained in log phase throughout the fitness assay by periodic dilution. G418 selection was maintained throughout the growth. Fitness, *w*_*i*_, was calculated as the slope of the log 2 relative abundance of the mutant to WT versus time over 6–10 time points spread over 6–8 WT doubling times. Selection coefficients, *s*, were calculated as 1 – *w*_*i*_ and normalized to the vector only control –(*s*_*i*_/*s*_vector_). Errors are propagated from the standard deviation of fitness measurements from three biological samples.

### 5.2 *Protein sequence for wildtype SsIGPS*

MSGSHHHHHHSSDIENLYFQGQRPIISLNERILEFNKSNITAIIAEYKRKSPSGLDVERDPIEYSKFMERYAVGLSILTEEKYFNGSYETLRKIASSVSIPILMKDFIVKESQIDDAYNLGADTVLLIVKILTERELESLLEYARSYGMEPLIEINDENDLDIALRIGARFIGINSRDLETLEINKENQRKLISMIPSNVVKVAESGISERNEIEELRKLGVNAFLIGSSLMRNPEKIKEFIL*.

### 5.3 *Yeast protein quantification by western blot*

Transformed yeast were collected and the associated protein levels of the SsWT and mutants quantified by Western blot. Whole yeast lysates were prepared using a LiAc and NaOH method.^48^ Samples were separated by electrophoresis and transferred to a membrane. A custom α‐His antibody (1:8,000) was purchased from Genscript to probe for His‐tagged IGPS. Normalization to an internal loading control, the housekeeping gene α‐tubulin (Abcam ab184970, 1:15,000), corrects for variability of the sample preparation and protein transfer. Fluorescently labeled secondary antibodies (LiCor 926‐32211, 926‐68073, 1:20,000) against the primary antibodies were used to detect protein levels using an Odyssey Imaging System. Errors are propagated from the standard deviation of measurements from three biological samples.

### 5.4 *Protein expression and purification*

The SsIGPS construct used for the fitness assay was cloned into the pGS21a expression vector using restriction sites, SacI, and BAMHI. *Escherichia coli* strain NiCo21(DE3) from NEB was transformed with the plasmids. Protein expression was induced with 1 mM IPTG for 4 hr. All proteins were isolated from inclusion bodies by dissolving the insoluble fraction of the cell lysate in 10 M urea and 10 mM imidazole, followed by sonication. Cell debris was removed by centrifugation and the soluble fraction bound to nickel resin for 1 hr at room temperature. The protein‐bound resin was washed with 10 column volumes of the same resuspension buffer. The protein was eluted with a step gradient of 20 and 250 mM imidazole. Pure fractions were pooled and dialyzed into 10 mM KPi, pH 7.2. The protein was further purified through a gradient elution off the Q‐column. Protein purity was confirmed by Coomassie‐staining of SDS‐PAGE gels.

### 5.5 *Circular dichroism structure analysis*

Far‐UV CD spectra were collected on a JASCO model J810 CD spectrophotometer. All samples were buffered with 10 mM KPi, pH 7.2 at 30°C. Measurements were taken in a 0.5 cm path length curvette with a bandwidth of 2.5 nm and a step size of 0.5 nm from 210 to 260 nm. Three spectra were collected, averaged, and buffer subtracted for each sample with a total averaging time of 3 s per wavelength. A protein concentration of ~3 μM was used for wavelength scans.

### 5.6 *Equilibrium unfolding*

Unfolded (~10 M buffered urea) and folded (10 mM KPi, pH 7.2 buffer) stocks of 3 μM protein were mixed to yield samples with titrated concentrations of urea from 0 to 9 M. Each sample was thoroughly mixed and equilibrated overnight in a 30°C incubator. CD spectra were collected as before. CD data were globally fit to a three‐state model, N ⇌ I ⇌ U, as a function of urea using in‐house Savuka software. Errors were propagated from the fit of the model.

### 5.7 *Enzyme kinetic assays*

Protein samples were made by combining to a final concentration of the following: 1 μM native protein and varying concentrations of CdRP [0–1,500 μM] in 10 mM KPi, pH 10 mM to a total volume size of 100 μl. The CdRP was synthesized by Adam Choi and Steven Pauff in the Stephan Miller laboratory at our institution. Twelve samples of with varying substrate concentrations were organized on a 96‐well black flat bottom plate. Prior to reaction initiation, proteins were incubated at 30°C. The chemical reaction was initiated upon addition of CdRP and the samples read immediately in a Tecan Infinite M1000 Pro plate reader, preheated to 30°C. A single orbital shake lasting 1 s at 306 rpm is applied before the first read. The fluorescence in each well was read for 80 kinetic cycles at an excitation of 280 nm with a 5 nm bandwidth and emission 348 nm with a 5 nm bandwidth. Errors are propagated from the standard deviation of measurements collected from three biological samples. The initial velocity is calculated as the RFU over time. The kinetic parameters were determined using the Michaelis–Menten Equation (1), where *v*_0_ is the initial velocity, *v*_m_ is the maximal rate of the reaction, [CdRP] is the concentration of substrate, and *K*_m_ is the Michaelis constant.

(1) $$v_{0}=v_{m}*\left(\;\frac{\left[\text{CdRP}\right]}{\left[\text{CdRP}\right]+K_{m}}\right)$$

### 5.8 *Flux dynamics model*

The flux dynamics model^28^, ^29^ describes the selection coefficient, *s*, using the product of the intracellular IGPS abundance [IGPS]_*i*_ and turnover number *k*_cat_ to determine the effective functional capacity and constants a, b that are dependent on environmental conditions:

(2) $$s=a*\left(\;\frac{{\left[\text{IGPS}\right]}_{i}*k_{\mathrm{cat}}}{b+k_{\mathrm{cat}}}\right)$$

## AUTHOR CONTRIBUTIONS

**Yvonne H. Chan:** Conceptualization; data curation; formal analysis; methodology; visualization; writing‐original draft; writing‐review and editing. **Konstantin B. Zeldovich:** Formal analysis; supervision; writing‐review and editing. **Charles R. Matthews:** Conceptualization; formal analysis; funding acquisition; supervision; writing‐review and editing.

## CONFLICT OF INTEREST

The authors declare no competing financial interests.

## Supporting information


**Data S1** **Table S1** describes the phenotype of each protein variant.
**Table S2** provides the thermodynamic and enzyme kinetic parameters of each mutant.
**Table S3** provides the interaction energy values of double mutants.
**Figure S1** demonstrates that most mutations are destabilizing.
**Figure S2** shows some mutants are more catalytically efficient than the wildtype protein.
**Figure S3** relates fitness as a function of the effective functional capacity.

## Data Availability

All data and scripts for data analyses are available at https://github.com/yvehchan/Allostery
