# Supplementary material for: An allosteric pathway explains beneficial fitness in yeast for long‐range mutations in an essential TIM barrel enzyme
Source: Protein Sci. 2020 Jul 20;29(9):1911–23. doi: 10.1002/pro.3911 (PMC7454521; doi:10.1002/pro.3911)
Supplement: Supplementary file 1 — Data S1 Table S1 describes the phenotype of each protein variant. Table S2 provides the thermodynamic and enzyme kinetic parameters of each mutant. Table S3 provides the interaction energy values of double mutants. Figure S1 demonstrates that most mutations are destabilizing. Figure S2 shows some mutants are more catalytically efficient than the wildtype protein. Figure S3 relates fitness as a function of the effective functional capacity. [file PRO-29-1911-s001.docx]

# Supplementary Tables

| Protein variant | Potential interaction of interest | Fitness phenotype | *s* | Relative conc |
| --- | --- | --- | --- | --- |
| SsWT |  |  | 0.00±0.00 | 1.0±0.00 |
| I45A | Non-canonical βα-hairpin clamp in module 1 | Wildtype-like | 0.02±0.01 | 1.08±0.21 |
| I45K | Non-canonical βα-hairpin clamp in module 1 | Beneficial | 0.17±0.01 | 2.02±0.44 |
| S70A | Alpha-helical residue with sidechain pointing within βαβ module 1 | Wildtype-like | -0.00±0.01 | 0.99±0.22 |
| M73A | Alpha-helical residue with sidechain pointing towards βαβ module 4 | Slightly deleterious | -0.12±0.01 | 1.01±0.22 |
| I107A | βα-hairpin clamp-Donor | Slightly beneficial | 0.03±0.01 | 1.65±0.34 |
| I107K | βα-hairpin clamp-Donor | Beneficial | 0.18±0.01 | 2.18±0.53 |
| I45A/S70A | Long range interaction within βαβ module 1 | Beneficial | 0.17±0.01 | 2.41±0.87 |
| I45A/M73A | Long range interaction within βαβ module 1 | Wildtype-like | -0.00±0.01 | 2.24±0.68 |
| I45K/S70A | Long range interaction within βαβ module 1 | Beneficial | 0.18±0.01 | 1.58±0.37 |
| I45K/I107A | Long range between βα-clamps from different βαβ modules | Beneficial | 0.18±0.01 | 2.65±0.60 |
| I45K/I107K | Long range between βα-clamps from different βαβ modules | Beneficial | 0.19±0.01 | 1.86±0.51 |
| S70A/I107K | Long range between α1 helix and βα2 clamp | Beneficial | 0.17±0.01 | 1.91±0.6 |

**Supplementary Table 1**

**Description of feature of interest, fitness phenotype, selection coefficient, and relative intracellular concentration for each IGPS variant**

| Protein variant | *s* | *(*θMRE222)/  ()WT | ΔGNI  (kcal·mol-1) | ΔGIU  (kcal·mol-1) | ΔΔGNI  (kcal·mol-1) | ΔΔGIU  (kcal·mol-1) | Vmax  (μM·s-1) | *k*cat (s-1) | KM  (μM) | Keff  (nM·s-1) | ΔΔG*k*cat (kcal·mol-1) |
| --- | --- | --- | --- | --- | --- | --- | --- | --- | --- | --- | --- |
| SsWT | 0.00±0.00 | 1.00 | 7.01±0.10 | 4.18±0.24 | - | - | 9.49±0.32 | 9.49±0.32 | 359.81±38.41 | 26.38±2.96 | - |
| I45A | 0.02±0.01 | 0.98 | 5.29±0.06 | 4.6±0.12 | 1.72±0.11 | -0.42±0.27 | 6.66±0.21 | 6.66±0.21 | 271.04±29.58 | 24.56±2.79 | 0.21±0.01 |
| I45K | 0.17±0.01 | 1.04 | 5.27±0.10 | 7.25±0.75 | 1.75±0.13 | -3.07±0.79 | 10.63±0.28 | 10.63±0.28 | 364.27±30.12 | 29.19±2.53 | -0.07±0.01 |
| S70A | -0.00±0.01 | 1.04 | 8.54±0.11 | 6.13±0.66 | -1.53±0.14 | -1.96±0.71 | 9.81±0.28 | 9.81±0.27 | 348.45±31.16 | 28.15±2.64 | -0.02±0.01 |
| M73A | -0.12±0.01 | 0.93 | 4.24±0.18 | 5.97±0.17 | 2.77±0.21 | -1.79±0.30 | 3.49±0.25 | 3.49±0.25 | 379.75±83.74 | 9.19±2.13 | 0.60±0.02 |
| I107A | 0.03±0.01 | 0.80 | 2.01±0.03 | 3.99±0.10 | 5.0±0.09 | 0.19±0.26 | 11.82±0.34 | 11.82±0.34 | 343.83±31.52 | 34.39±3.30 | -0.13±0.01 |
| I107K | 0.18±0.01 | 0.97 | 3.06±0.04 | 4.78±0.08 | 3.95±0.10 | -0.6±0.26 | 13.96±0.23 | 13.96±0.23 | 399.70±20.06 | 34.92±1.85 | -0.23±0.01 |
| I45A/S70A | 0.17±0.01 | 0.95 | 4.89±0.14 | 4.75±0.28 | 2.12±0.17 | -0.57±0.37 | 10.23±0.39 | 10.23±0.39 | 462.29±50.15 | 22.14±2.55 | -0.05±0.01 |
| I45A/M73A | -0.00±0.01 | 0.76 | 1.86±0.23 | 6.19±0.30 | 5.15±0.25 | -2.02±0.39 | 6.56±0.38 | 6.56±0.38 | 440.83±73.40 | 14.88±2.62 | 0.22±0.02 |
| I45K/S70A | 0.18±0.01 | 1.07 | 10.58±0.16 | 6.18±0.61 | -3.57±0.18 | -2.0±0.66 | 11.80±0.48 | 11.80±0.48 | 507.04±55.71 | 23.28±2.73 | -0.13±0.02 |
| I45K/I107A | 0.18±0.01 | 0.97 | 3.54±0.08 | 3.72±0.09 | 3.48±0.12 | 0.45±0.26 | 11.73±0.36 | 11.73±0.36 | 444.73±39.42 | 26.37±2.47 | -0.13±0.01 |
| I45K/I107K | 0.19±0.01 | 0.91 | 3.65±0.06 | 3.47±0.06 | 3.37±0.11 | 0.7±0.25 | 13.14±0.19 | 13.14±0.19 | 354.82±16.41 | 37.03±1.78 | -0.19±0.01 |
| S70A/I107K | 0.17±0.01 | 0.91 | 5.39±0.07 | 3.34±0.13 | 1.62±0.11 | 0.84±0.28 | 14.60±0.43 | 14.60±0.43 | 421.73±37.06 | 34.61±3.21 | -0.26±0.01 |

**Supplementary Table 2**

**Thermodynamic and enzyme kinetic parameters of each mutant**

| Protein variant | δ*s* | δNI (kcal·mol-1) | δIU (kcal·mol-1) | δ*k*cat (kcal·mol-1) |
| --- | --- | --- | --- | --- |
| I45A/S70A | 0.151+/-0.01 | 1.93±0.24 | 1.81±0.85 | -0.24±0.02 |
| I45A/M73A | 0.095+/-0.01 | 0.67±0.34 | 0.2±0.56 | -0.59±0.03 |
| I45K/S70A | 0.009+/-0.01 | -3.78±0.26 | 3.03±1.25 | -0.04±0.02 |
| I45K/I107A | -0.017+/-0.01 | -3.27±0.20 | 3.34±0.87 | 0.07±0.02 |
| I45K/I107K | -0.162+/-0.01 | -2.33±0.20 | 4.38±0.87 | 0.11±0.02 |
| S70A/I107K | -0.004+/-0.01 | -0.8±0.21 | 3.39±0.80 | -0.01±0.02 |

**Supplementary Table 3**

**Interaction energy of double mutants**

# Supplementary Figures


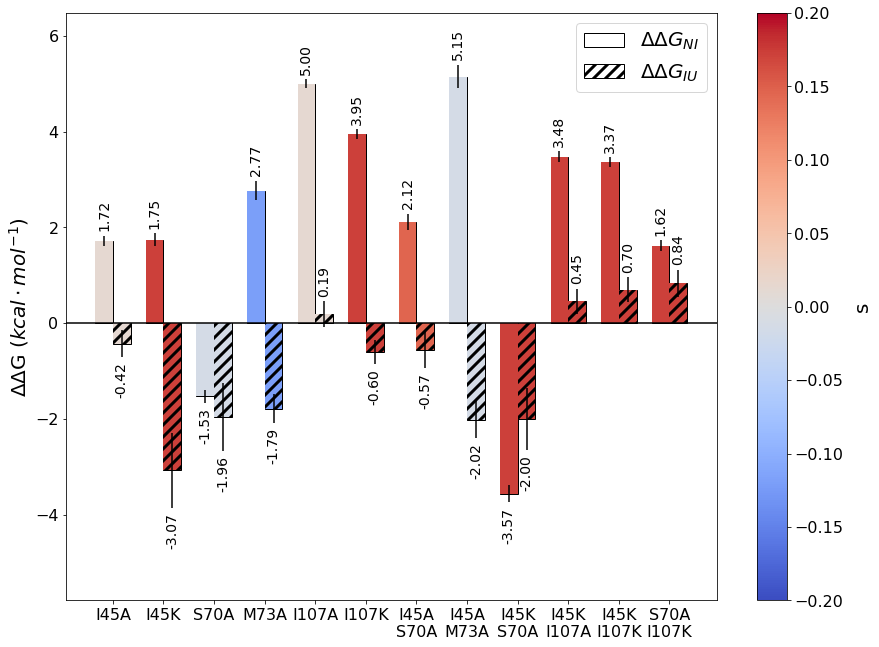

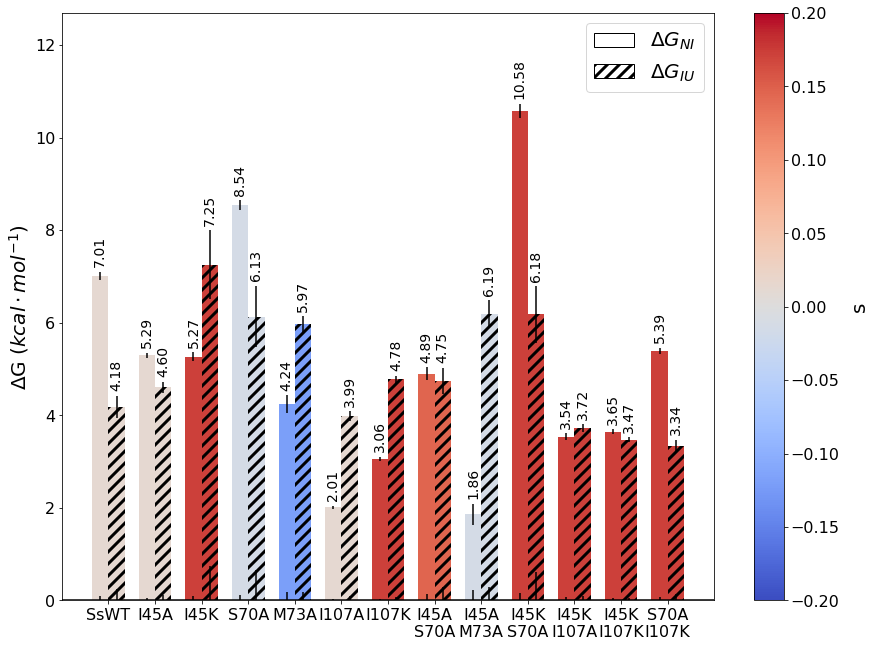


**Supplementary Figure 1**

**Most mutations are destabilizing.**

**(Top)** The free energy differences of unfolding for the N to I transition, ΔG°NI (solid fill), and for the I to U transition, ΔG°IU (hatched fill) are graphically represented in a bar graph. **(Bottom)** Changes in protein stability induced by the mutations, ΔΔG°NI and ΔΔG°IU, were determined as the difference in free energy between SsWT and the SsIGPS variant for the respective NI and IU transitions. Bars are colored by selection coefficient. Errors are propagated from the fit of the model.


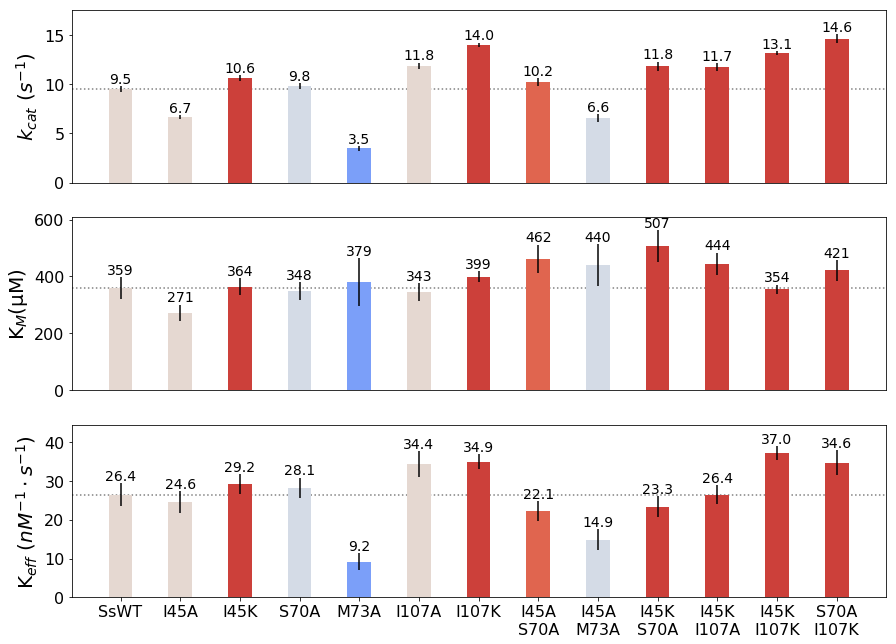


**Supplementary Figure 2**

**Several SsIGPS mutants were more catalytically efficient than SsWT.**

The kinetic parameters were plotted as bargraphs: (top) *kcat*, (middle) KM, (bottom) Keff. Several mutants showed greater catalytic efficiency compared to SsWT. Bars are colored by the selection coefficients. The gray dotted line is a visual guide for the value associated with SsWT. Errors are propagated from the standard deviation of measurements collected from three biological samples.


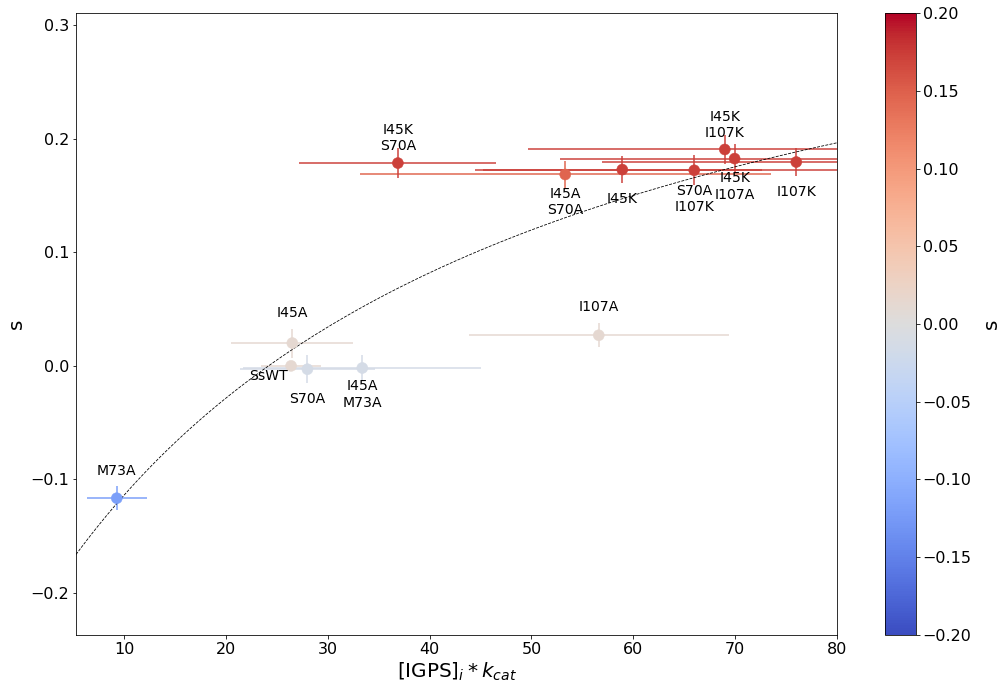


**Supplementary Figure 3**

**Fitness is a function of the effective functional capacity that depends on stability and activity.**

Selection coefficients were plotted as a function of effective functional capacity, [IGPS]i * *k*cat , demonstrating a non-linear positive relationship. Markers are colored by selection coefficient. Mutants are individually labeled near the markers. The fit is denoted by the black dashed line (See Methods, Equation 2). Errors are propagated from the standard deviation of measurements collected from three biological samples for the enzymatic assay and for protein quantification in the X-axis and for the selection coefficient for the Y-axis.
